# Clinical implications of pachyvessels in polypoidal choroidal vasculopathy

**DOI:** 10.1186/s12886-020-01443-8

**Published:** 2020-04-29

**Authors:** Nobuo Ijuin, Hiroki Tsujinaka, Hiromasa Hirai, Hironobu Jimura, Shigeya Nakao, Mariko Yamashita, Tomo Nishi, Tetsuo Ueda, Nahoko Ogata

**Affiliations:** 1 Department of Ophthalmology, Nara City Hospital, Nara, Japan; 2 Department of Ophthalmology, Nara Medical University, 840 Shijo-cho, Kashihara, Nara, 634-8522 Japan

**Keywords:** Polypoidal choroidal vasculopathy, Pachychoroid, Pachyvessels

## Abstract

**Background:**

Polypoidal choroidal vasculopathy (PCV) is one of the disorders within the pachychoroid spectrum diseases. The presence of pachyvessels is one of the characteristics of pachychoroid disorders. However, the relationship between the presence of pachyvessels and the clinical characteristics of PCV eyes has not been determined. The purpose of this study was to determine the relationship between the presence of choroidal pachyvessels and the clinical characteristics of eyes with PCV.

**Methods:**

The medical records of patients who were diagnosed with PCV and were treatment-naïve were reviewed. Fluorescein and indocyanine green angiography, fundus photography, spectral domain optical coherence tomography (SD-OCT), and enhanced depth imaging OCT (EDI-OCT) were used to obtain images of the choroid. The presence of pathologically dilated outer choroidal vessels, pachyvessels, was determined by ICGA images. These pachyvessels were confirmed to correspond with the large choroidal vessels in the EDI OCT images. The PCV eyes were divided into two groups based on the presence or absence of pachyvessels and clinical features and subfoveal choroidal thickness (SFCT) were evaluated between the two groups.

**Results:**

Eighty-six eyes of 84 patients with PCV were evaluated. Pachyvessels were detected in 48 eyes (55.8%). The mean SFCT was 203.9 ± 83.9 μm in all 86 eyes, and it was significantly thinner in eyes with pachyvessels (+) than without pachyvessels (−) (183.2 ± 58.4 μm vs 230.2 ± 103.1 μm; *P* = 0.01). The differences in the incidence of subretinal fluid, pigment epithelial detachments, and hemorrhages between the two groups were not significant. However, the PCV eyes in pachyvessels (+) group with hemorrhage had the thinnest choroid (*P* = 0.047). The choroidal features of the fellow eyes were similar to those of the PCV affected eyes, that is, the fellow eyes in pachyvessels (+) group had pachyvessels and the fellow eyes in pachyvessels (−) group did not have pachyvessels.

**Conclusions:**

Pachyvessels were presented 55.8% in eyes with PCV, and these eyes had the thin SFCT. The presence of pachyvessels and attenuation of the inner choroid were probably due to the pathological changes in the eyes with PCV.

## Background

Polypoidal choroidal vasculopathy (PCV) was first described by Yannuzzi et al. [1], and it was considered to be a subtype of neovascular age-related macular degeneration (AMD). PCV is characterized by polypoidal lesions which are detected as dilations of the terminals of branching vascular networks [1, 2]. In addition, the pachychoroidal spectrum disorders are characterized by alterations to the choroid which may be manifested as increased choroidal thickness, presence of pathologically dilated choroidal vessels in Haller layer, i.e., pachyvessels, and regional choroidal hyperpermeability [3–5]. The choroidal thickening and choroidal hyperpermeability indicates [6, 7] that PCV is within the pachychoroidal spectrum disorders.

The mean subfoveal choroidal thickness (SFCT) has been shown to be increased in eyes with PCV, however there was pronounced interindividual variability with a wide range of thicknesses [8–11]. The thickness of the choroid is not always increased in eyes with PCV [8, 10, 12]. The pachyvessels are usually associated with a thinning of the overlying choriocapillaris in eyes with PCV [13]. This feature was even observed in eyes with normal-to-thin choroidal PCV [8, 10, 12]. Although the term pachychoroid was originally coined to reflect a choroidal thickening, subsequently the choroidal thickness was found not to be the most important criterion for defining pachychoroid disorders [14].

The mechanism by which pachychoroid and/or pachyvessels might predispose the eye to the development of PCV has not been conclusively determined. However, a relative ischemia due to a thinning of the choriocapillaris and/or a chronic mechanical disruption of Bruch’s membrane have been proposed [3, 4, 13]. A thinning of the choriocapillaris and Sattler’s layer with or without retinal pigment epithelium (RPE) abnormalities may accompany the pachyvessels [4, 5, 15]. In spite of findings in the earlier studies, how choroidal changes attribute to clinical characteristics of PCV has not been well known.

Thus, the purpose of this study was to determine the incidence and characteristics of PCV eyes with pachyvessels. To accomplish this, we determined the SFCT and reviewed the clinical characteristics such as the presence of subretinal fluid, pigment epithelial detachments, and hemorrhages in eyes with and without pachyvessels.

## Methods

This retrospective study was performed in the Department of Ophthalmology at Nara Medical University. The study was approved by the hospital’s institutional review board (IRB. No.2107) and was conducted according to the Declaration of Helsinki.

We reviewed the electronic medical records of patients who were examined and diagnosed with treatment- naïve PCV at the Nara Medical University Hospital from November 2012 through May 2018. All the patient had undergone a comprehensive ocular examination that included measurements of the best-corrected visual acuity (BCVA), intraocular pressures, and refractive errors (spherical equivalent) by autorefractometry (KR8100, RM8900, Topcon Corporation, Tokyo, Japan). In addition, color fundus photography (TRC-NW8; Topcon Corporation, Tokyo, Japan), fluorescein angiography (FA), and indocyanine green angiography (ICGA) with a confocal laser scanning ophthalmoscope (HRA2; Heidelberg Engineering, Heidelberg, Germany), and spectral domain optical coherence tomography (SD-OCT, Spectralis HRA &OCT; Heidelberg Engineering, Heidelberg, Germany) with the enhanced depth imaging (EDI) program were performed.

The PCV was primarily diagnosed by the presence of branching vascular networks and terminating polypoidal lesion(s) in the ICGA images as reported [2]. Both the affected eye and fellow eye were studied. The exclusion criteria were: myopia exceeding − 6.0 diopters (D) or axial length > 26 mm, presence of subretinal hemorrhages that could obscure the choroidal vessels, and presence of other ocular and systemic diseases, including diabetic retinopathy, retinal detachment, macular hole, uveitis, tumors, and glaucoma. Eyes with prior treatments that can cause significant changes to the choroid such as anti-vascular endothelial growth factor (anti-VEGF) therapy, photodynamic therapy, laser photocoagulation, and use of intraocular, periocular or systemic corticosteroids were excluded. Eyes with severe media opacities that could degrade the quality of the images such as cataracts and vitreous opacities and eyes with severe subretinal hemorrhages or vitreous hemorrhages were also excluded.

### Subfoveal choroidal thickness measurements

The subfoveal choroidal thickness (SFCT), was defined as the distance between Bruch’s membrane and the choroid-scleral border at the fovea. This was manually measured by independent AMD specialists (M.Y. and H.H.) using the horizontal and vertical line scans intersecting at the center of the fovea of the SD-OCT images with the EDI program according to the previous reports [8–12, 16, 17]. The inter-observer reproducibility of SFCT was evaluated using intraclass correlation coefficient (ICC), and the ICC was excellent (ICC = 0.92).

### Assessments of pachychoroid spectrum and choroidal pachyvessels

Two independent AMD specialists (M.Y. and H.H.) performed all of the diagnosis based on the criteria of pachychoroid spectrum disorders [18]. The supervising grader (N.O.) was consulted when the other two graders disagreed and made the final diagnosis.

The presence of pathologically dilated outer choroidal vessels, pachyvessels, was determined by choroidal vessels in the ICGA images according to the previous reports [12, 16, 17]. We confirmed these pachyvessels corresponded with the large choroidal vessels in the EDI OCT and OCT raster scan images (6X6 mm) [3, 5, 17].

Choroidal vascular hyperpermeability was determined by detecting multifocal hyper fluorescent areas with blurred margins that expanded during the late phase of ICGA.

PCV eyes were divided into two groups based on the presence of pachyvessels, that is, pachyvessels positive (+) group and pachyvessels negative (−) group for analysis of the clinical characteristics.

The types of lesion, the presence of subretinal fluid (SRF), pigment epithelial detachment (PED), and subretinal hemorrhage were determined by examining the color fundus photographs and SD-OCT images.

### Statistical analyses

Statistical analyses were performed with the SPSS for Windows (version 22.0.1; SPSS Inc., Chicago, IL). Unpaired *t*-tests and one-way analysis of variance (ANOVA) were used to compare continuous variables between the two groups [16]. Categorical variables of the two groups were compared using chi-square tests. Analysis of covariance was used for adjustment of the variables. A *P* value < 0.05 was considered statistically significant.

## Results

Eighty-six eyes of 84 PCV patients were evaluated. The baseline characteristics are summarized in Table 1. The mean age of the patients was 73.9 ± 7.9 years (± standard deviation (SD)) with a range of 53 to 89 years. There were 63 men (74.1 ± 7.2 years, 75%) and 21 women (71.8 ± 9.7 years). Two patients (one man and one woman) had PCV in both eyes. All patients were Japanese.

**Table 1:** Basic Characteristics of eyes with PCV

	Affected eye	pachyvessels (+)	pachyvessels (−)
Eye, (patient, n)	86 (84)	48 (47)	38 (37)
Men/Women (men %)	63/21 (75)	33/14 (70.2)	30/7 (81.1)
male eyes, n (%) female eyes, n (%)	64 22	33 (51.6) 15 (68.2)	31 (48.4) 7 (31.8)
Age (SD), years	73.9 (7.9)	73.9 (7.9)	73.2 (8.0)
men/women	74.1(7.2)/71.8 (9.7)	75.0 (8.6)/71.2 (10.2)	73.2 (7.9)/73.0 (9.2)
BCVA (SD), logMAR units	0.38 (0.45)	0.35 (0.40)	0.44 (0.50)

The differences between eyes with pachyvessels (+) group and without pachyvessels (−) group. Data of age and BCVA are expressed as the means ± SD

Basic characteristics of SFCT in this study were shown in the supplementary figure.

The SFCT tended to be thinner depending on aging. The SFCT was not significant different between genders and with or without retinal hemorrhage.

Pachyvessels were detected in 48 eyes of 47 patients (55.8%; mean age, 73.9 ± 7.9 years), and 33 eyes were of male patients (70.2%; 75.0 ± 8.6 years) and 15 eyes were of 14 female patients (29.8%; 71.2 ± 10.2 years). Pachyvessels were not detected in 38 eyes of 37 patients (44.2%; 73.2 ± 8.0 years), and 31 eyes were of male patients (81.1%; 73.2 ± 7.9 years) and 7 eyes were of female patients (18.9%; 73.0 ± 9.2 years).

The difference in the ages between the eyes with or without pachyvessels was not significant. Pachyvessels were present in 51.6% of the men and 68.2% of the women *(P* > 0.05).

The BCVA was not significantly different between the eyes with and without pachyvessels (0.35 ± 0.40 vs 0.44 ± 0.50 logarithm of the minimum angle of resolution units; Table 1).

The mean SFCT was 203.9 ± 83.9 μm with a range of 50 to 471 μm in all 86 eyes, and the SFCT in pachyvessels (+) group was significantly thinner than that in the pachyvessels (−) group (183.2 ± 58.4 μm vs 230.2 ± 103.1 μm, *P* = 0.01; Table 2).

**Table 2:** Clinical Characteristics of PCV eyes with or without pachyvessels

	Affected eye	pachyvessels (+)	pachyvessels (−)	***P***
Eye,n(%)	86	48 (55.8)	38 (44.2)	
SFCT (SD), μm	203.9 (83.9)	183.2 (58.4)	230.2 (103.1)	0.010*
Clinical Findings				
Subretinal fluid, n (%)	59 (68.6)	32 (66.7)	27 (71.1)	
SFCT (SD), μm	205.3 (97.0)	189.9 (55.9)	245.3 (105.2)	0.014*
Pigment epithelial detachment, n (%)	30 (34.9)	17 (35.4)	13 (34.2)	
SFCT (SD), μm	197.0 (84.7)	173.6 (99.0)	205.6 (87.8)	0.127
Hemorrhage, n (%)	14 (16.3)	6 (12.5)	8 (21.1)	
SFCT (SD), μm	174.2 (55.1)	147.6 (82.2)	202.8 (92.3)	0.047*

Clinical findings were overlapped counted. The mean SFCT was expressed as SFCT (SD). The mean SFCT was significantly thinner in eyes with pachyvessels (+) than that of pachyvessels (−) (*P* = 0.010). *:*P* < 0.05

SRF was observed in 59 of 86 (68.6%) eyes including 32 of 48 (66.7%) eyes in the pachyvessels (+) group and 27 of 38 (71.1%) eyes in pachyvessels (−) group (*P* > 0.05). The mean SFCT in eye with SRF was 205.3 ± 97.0 μm which was significantly thinner in the pachyvessels (+) group (189.9 ± 55.9 μm) than in the pachyvessels (−) group (245.3 ± 105.2 μm, *P* = 0.014; Table 2).

A PED was observed in 30 of 86 eyes (34.9%) including 17 of 48 (35.4%) eyes in the pachyvessels (+) group and 13 of 38 (34.2%) eyes in the pachyvessels (−) group (*P* > 0.05). The mean SFCT in eyes with a PED was 197.0 ± 84.7 μm, and the difference between the pachyvessels (+) group (173.6 ± 99.0 μm) and the pachyvessels (−) group (205.6 ± 87.6 μm) was not significant (*P* = 0.127; Table 2).

Retinal and subretinal hemorrhages were observed in 14 of 86 eyes (16.3%) including 6 of 48 (12.5%) in eyes of the pachyvessels (+) group and 8 of 38 (21.1%) eyes of the in pachyvessels (−) group. The mean SFCT in all eye with hemorrhages was 174.2 ± 55.1 μm. However, it was significantly thinner in the pachyvessels (+) group (147.6 ± 82.2 μm) than that in the pachyvessels (−) group (202.8 ± 92.3 μm, *P* = 0.047; Table 2).

As for fellow eyes, pachyvessels were detected in 38 of the 82 (46.3%). In the pachyvessels (+) group, 36 of 46 (78.3%) fellow eyes had pachyvessels, whereas in pachyvessels (−) group, only 2 of 36 eyes (5.6%) had pachyvessels. These results indicated that the fellow eyes in pachyvessels (+) group had pachyvessels (*P*<0.01, chi-square test).

The mean SFCT of fellow eyes with pachyvessels (193.0 ± 73.7 μm) was significantly thinner than that without pachyvessels (234.4 ± 89.3 μm, *P* = 0.028; Table 3).

**Table 3:** Presence of pachyvessels and SFCT in fellow eyes compared to that of affected eyes

		Affected eye *N* = 82 SFCT (SD) μm 204.6 (85.3)		
		pachyvessels (+) *N* = 46 187.3 (71.5)	pachyvessels (−) *N* = 36 249.3 (87.7)	*P* < 0.001***
**Fellow eye** *N* = 82 SFCT (SD) μm 215.2 (84.5)	**pachyvessels (+)** *N* = 38 (46.3%) 193.0 (73.7)	36 (78.3%) 189.9 (74.6)	2 (5.6%) 246.3 (3.2)	0.3
	**pachyvessels (−)** *N* = 44 (57.7%) 234.4 (89.3)	10 (21.7%) 177.2 (60.3)	34 (94.4%) 249.5 (90.3)	0.029*

The mean SFCT in the fellow eyes with pachyvessels was significantly thinner than that of without pachyvessels (*P* = 0.028). The fellow eyes in pachyvessels positive (+) group have pachyvessels (*P* = 1.28 × 10–9, chi-square test). The mean SFCT was expressed as SFCT (SD). *:*P* < 0.05

### Representative cases with pachyvessels (+) and pachyvessels (−)

#### Case 1

**Fig. 1:**
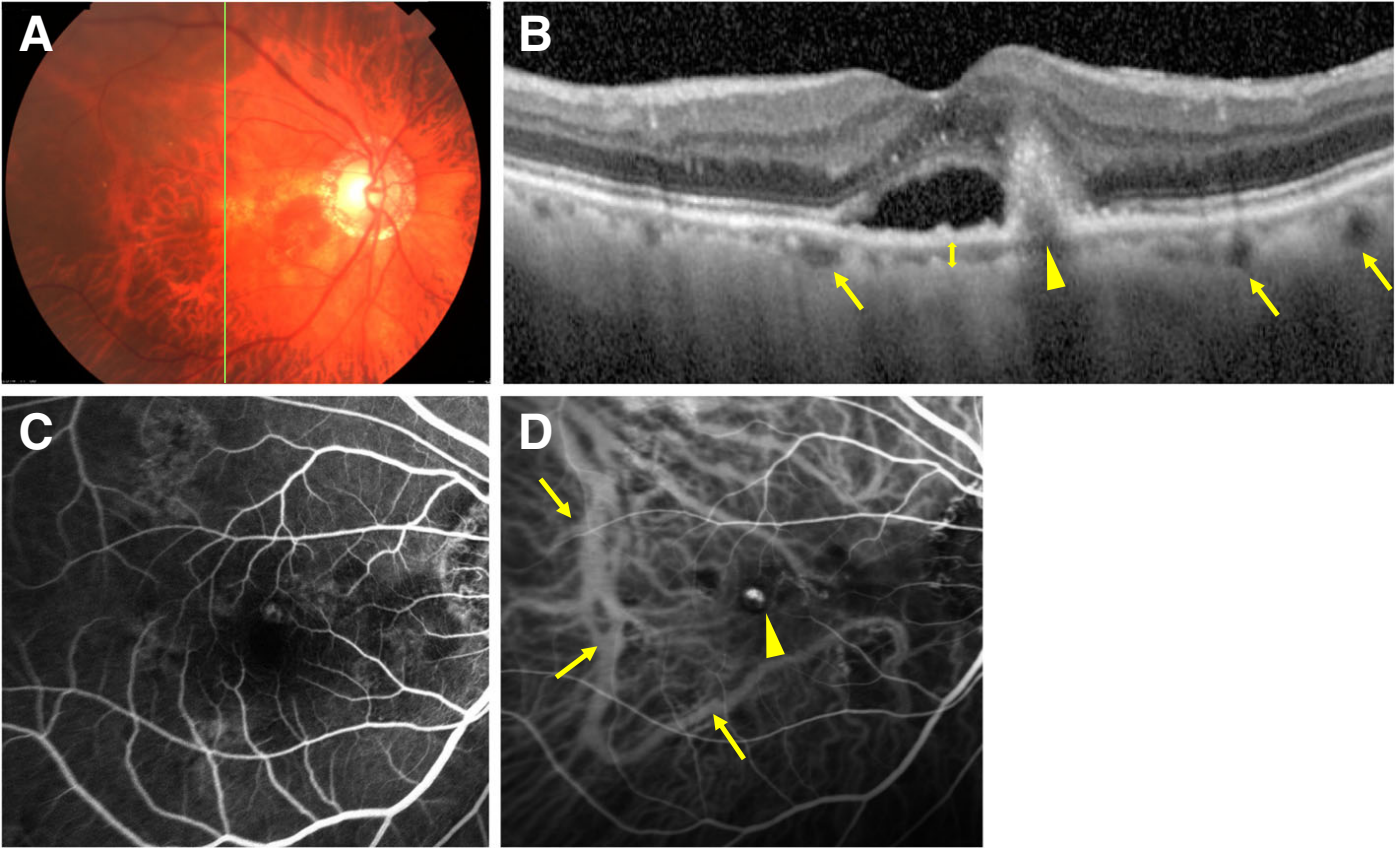
Case of polypoidal choroidal vascularization (PCV) with pachyvessels (+). A mid-eighties person whose BCVA was 6/20 at the baseline. **a**. Fundus color photograph. **b**. EDI-OCT. OCT image shows a thin choroid and the subfoveal choroidal thickness (SFCT) was 50.0 μm. Arrowhead indicates a polyp and arrows indicate pachvessels. **c**. Fluorescein angiographic image at 15.3 s. **d**. Indocyanine angiography at 57 s showing a polyp. Arrowhead indicates polyp, arrows indicate pachyvessels

A mid-eighties person with PCV in the right eye was examined (Fig. 1a). The BCVA was 20/100 in the right eye. A polyp (arrowhead) and pachyvessels (arrows) can be seen in the ICGA images (Fig. 1d). The OCT image showed a thin choroid and the SFCT was thin at 50 μm (Fig. 1b).

#### Case 2

**Fig. 2:**
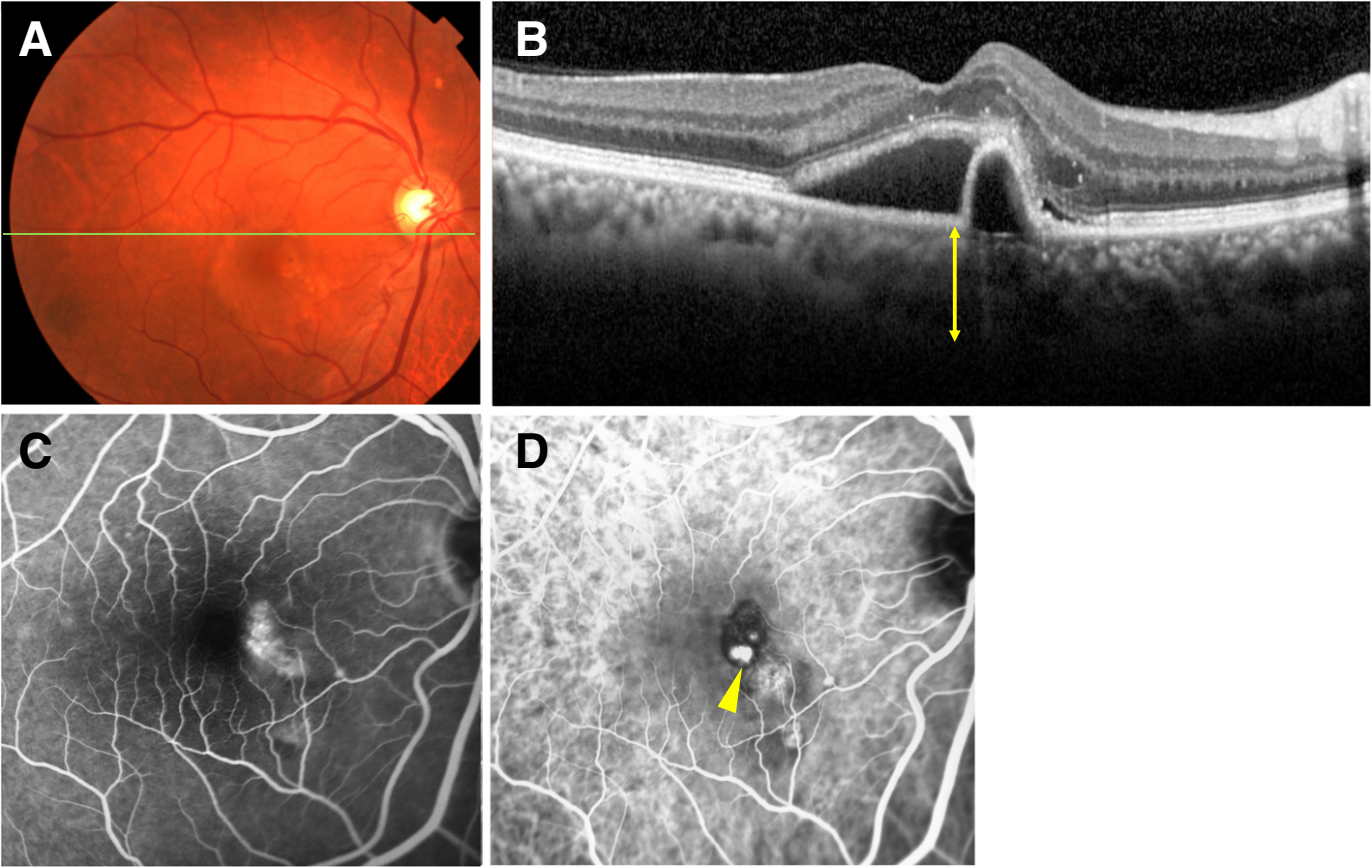
Case of PCV without pachyvessels (−). A mid-sixties person whose baseline BCVA was 18/20. **a**: Fundus color photograph. **b**: EDI-OCT image shows a thick choroid. The SFCT was 299 μm. **c**: Fluorescein angiography at 45.5 s. **d**: ICGA at 24 s. Arrowhead indicates a polyp. Pachvessels are not present

A mid-sixties person with PCV in the right eye was examined (Fig. 2a). The BCVA in the right eye was 20/25. A polyp (arrowhead) was detected by ICGA but pachyvessels were not present (Fig. 2d). The choroid was thick and the SFCT was 299 μm.

## Discussion

The pachyvessels are a well-established choroidal features in eyes with pachychoroidal spectrum disorders [5, 12, 13, 17–19].

Our results showed that pachyvessels were detected in 55.8% of eyes with PCV which is much lower than that reported in 85% [16]. The reason for this difference may be due to differences in the ethnicity and race. Although there are no objective size criteria for pachyvessels, the higher incidence of pachyvessel in PCV supports the role of choroidal vessel dilatations in the pathogenesis of these diseases.

The results of previous study of PCV showed that the distribution of the subfoveal choroidal thickness was bimodal with peaks at 170 and 390 μm [12]. This suggested that PCV may consists of two overlapping phenotypes with one having markedly increased subfoveal choroidal thickness.

We found that the mean SFCT was significantly thinner in the pachyvessels (+) group than in the pachyvessels (−) group. It has been supposed that this may occur when the luminal volume increases secondary to the outer choroidal vessel dilation is offset by a reduction in tissue volume from a concurrent atrophy of the inner choroidal vasculature [3, 17]. Thus, it is possible for an eye to have normal or even subnormal choroidal thickness but still have the pachychoroid disease phenotype [3].

Pachyvessels and attenuation of inner choroid seem to be key features of the pachychoroidal changes in eyes with PCV. A loss of the choriocapillaris may produce a relatively ischemic environment leading to the overexpression of angiogenic factors. While the exact pathogenic mechanisms for these choroidal changes remain to be validated, some plausible hypotheses including an engorgement of the vortex veins, choroidal vascular hyperpermeability, and choroidal venous hypertension can be considered [7, 20, 21].

The results of a recent study showed that pachyvessels were associated with choriocapillaris flow impairment by location and size [22]. Whether the occurrence of dilated Haller pachyvessels is a primary event or secondary to the inner choroidal attenuation is still under investigation. It is possible that ischemic, inflammatory, or involutional insults to the inner choroidal circulation can result in a loss of the inner choroid leading to arteriovenous shunting with the resultant venous dilation.

Although the choroidal changes are possibly involved in the pathogenesis of PCV, it remains unclear whether the PCV phenotypes with varying choroidal thickness have similar neovascular processes and clinical characteristics. These dilated large choroidal vessels under the disease foci can be associated with disease development or progression.

We investigated the association between the pachyvessels and the SFCT, and between the pachyvessels and the clinical characteristics of eyes with PCV. There were no significant differences in the incidence of SRF, PED, and hemorrhages between the pachyvessels (+) group and pachyvessels (−) group confirming earlier results [12]. They found that there was not a significant difference in the central retinal thickness and presence of PED and hemorrhages between the thin SFCT group and thick SFCT group.

However, our results showed that the PCV eyes with SRF and hemorrhages in the pachyvessels (+) group had significantly thinner SFCT than that of pachyvessels (−) group especially the eyes with hemorrhages. Although there was not a significant difference in SFCT between eyes with SRF, PED, and hemorrhages, this may have been because of the small number of eyes studied. The PCV eyes in pachyvessels (+) group with hemorrhages had the thinnest choroid. These findings suggest that the choroidal changes are possibly involved in the pathogenesis of PCV and the thin choroid may likely be the cause of the hemorrhages because of the ischemic condition.

It has been reported that an increased SFCT and choroidal vascular hyperpermeability are associated with poor outcomes for anti-VEGF treatment [7, 23–25]. Our results suggested that the thin choroid may be a risk of hemorrhages.

An earlier study reported that some of the fellow eyes in patients with PCV had pachyvessels in the absence of neovascular or polypoidal sequelae [26]. This suggested that the pachychoroid features were not a reaction to the neovascularization.

Our findings showed that pachyvessels were detected in 46.3% of the fellow eyes and most of the fellow eyes (78.3%) in pachyvessels (+) group had pachyvessels. This is consistent with published observations in non-Asian cohorts [12, 13]. Our data also suggested that the fellow eyes had a potentially of developing pathogenic changes of the choroid.

This study has several limitations including its retrospective and noncomparative design. The mean SFCT of all PCV in this cohort was thinner than that of expected. It can be affected by excluding eyes with severe submacular hemorrhages or vitreous hemorrhages. Hemorrhagic PCV may not have characteristics similar to exudative PCV. There is no consensus for defining the thickness of the subfoveal choroid. The thickness can be influenced by various factors, such as age, axial length, and refractive error [27]. Furthermore, there are no objective size criteria or definition for pachyvessels. Further investigations may define these vessels in more detail.

## Conclusions

pachyvessels were present in 55.8% of eyes with PCV, and these eyes had a significantly thinner SFCT. The presence of pachyvessels and attenuation of the inner choroid seem to be due to the pathological changes in eyes with PCV.

## Supplementary information

**Additional file 1 **F**igure** S4 Basic characteristics of SFCT in this study

## Data Availability

The all data used to support the findings of this study are available from the. corresponding author upon request.

## Abbreviations

PCV: Polypoidal choroidal vasculopathy
SD-OCT: Spectral domain optical coherence tomography
EDI-OCT: Enhanced depth imaging optical coherence tomography
SFCT: Subfoveal choroidal thickness
AMD: Age-related macular degeneration
RPE: Retinal pigment epithelium
FA: Fluorescein angiography
ICGA: Indocyanine green angiography
anti-VEGF: Anti-vascular endothelial growth factor
ICC: Intraclass correlation coefficient
SRF: Subretinal fluid
PED: Pigment epithelial detachment
SD: Standard deviation
