# Supplementary figures and images for: Clinical implications of pachyvessels in polypoidal choroidal vasculopathy
Source: BMC Ophthalmol. 2020 Apr 29;20:170. doi: 10.1186/s12886-020-01443-8 (PMC7191784; doi:10.1186/s12886-020-01443-8)

## Slide 1
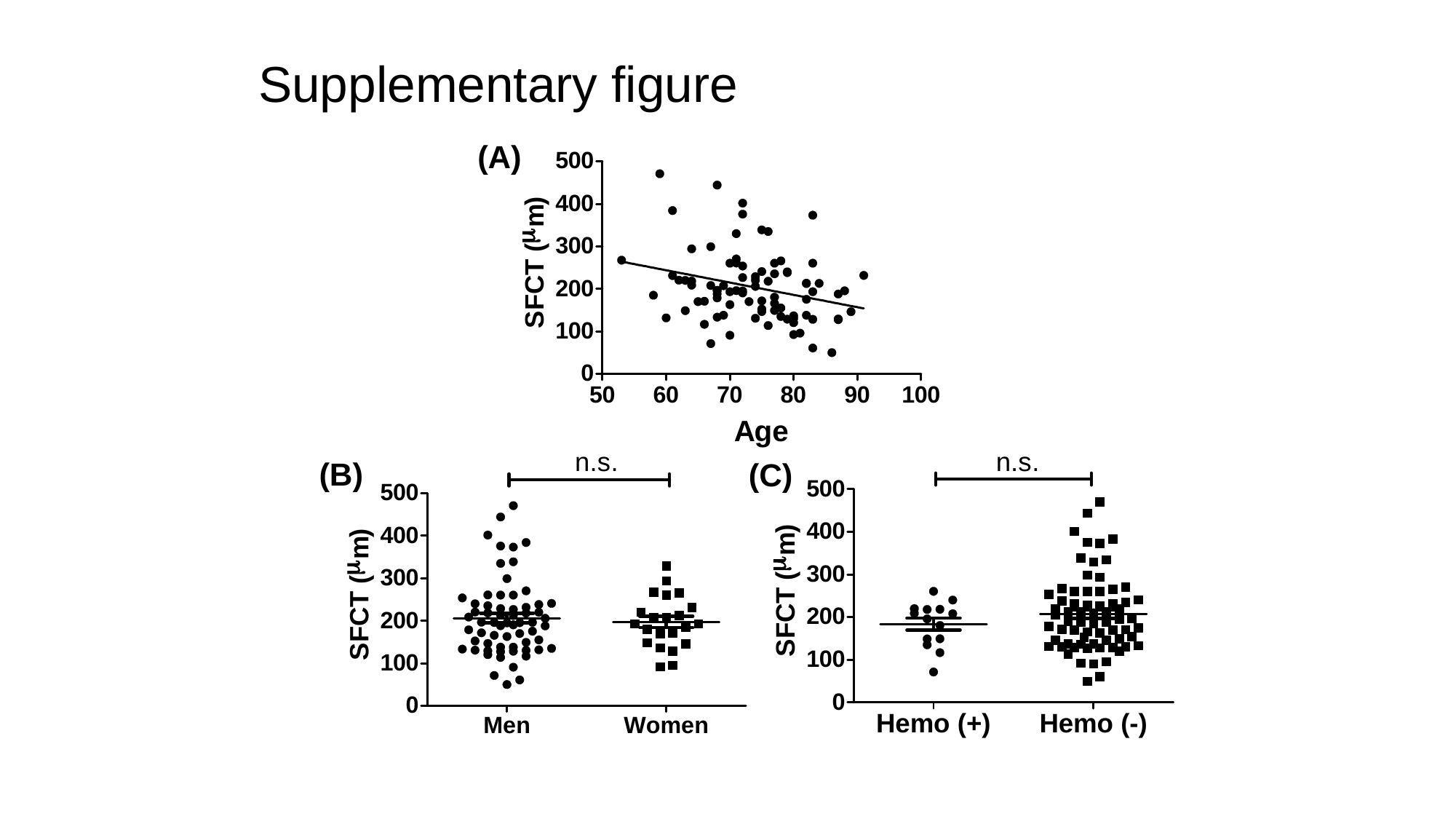

Supplementary figure
(A)
(B)
(C)

Supplement: Supplementary file 1 — Additional file 1 Figure S4 Basic characteristics of SFCT in this study [file 12886_2020_1443_MOESM1_ESM.pptx]
